# Daily consumption of milk fat globule membrane plus habitual exercise improves physical performance in healthy middle-aged adults

**DOI:** 10.1186/s40064-015-0896-8

**Published:** 2015-03-10

**Authors:** Noriyasu Ota, Satoko Soga, Tadashi Hase, Akira Shimotoyodome

**Affiliations:** Biological Science Research Laboratories, Kao Corporation, 2606 Akabane, Ichikai-machi, Haga-gun, Tochigi, 321-3497 Japan

**Keywords:** Agility, Aging, Milk fat globule membrane, Motor unit, Muscle cross-sectional area, Physical performance

## Abstract

Our recent studies demonstrated that habitual exercise plus dietary supplementation with milk fat globule membrane (MFGM) improved endurance capacity and muscle function by stimulating neuromuscular development in mice. The aim of this study was to investigate the efficacy of dietary MFGM supplementation plus habitual exercise on the physical performance of middle-aged Japanese adults in a pilot randomized, double-blind, placebo-controlled trial. Forty-four subjects (men, n = 22; women, n = 22) were randomly assigned into two groups: one received placebo tablets (placebo group, n = 22 [men, n = 11; women, n = 11]), while the other received MFGM tablets (MFGM group, n = 22 [men, n = 11; women, n = 11]). The subjects ingested either MFGM (1 g/day) or placebo (1 g/day of whole milk powder) tablets every day for the 10-week study period and engaged in an exercise training program twice per week. A physical function test was performed at baseline and at 5 and 10 weeks. A significant group-by-time interaction was found for the side step test, muscle cross-sectional area (CSA), and muscle fiber conduction velocity (MFCV). In the placebo group, there were no significant intragroup differences. In the MFGM group, side step score and muscle CSA were significantly greater at 10 weeks compared to the baseline, and MFCV was significantly higher than that in the placebo group at 10 weeks. The changes in percentage of the side step score, muscle CSA, and MFCV in the MFGM group were significantly higher than in the placebo group at 10 weeks. These results suggest that daily MFGM ingestion combined with regular exercise might enhance physical performance such as agility in middle-aged adults.

## Background

Age-related muscle wasting and weakness are highly associated with adverse effects on quality of life and life expectancy in elderly people (Evans 1995). Therefore, it is important to develop strategies to attenuate the decline in muscular mass and strength. Age-related decreases in thigh muscle mass begin in the fourth decade of life in healthy humans (Nair 2005). Between 50 and 80 years of age, the number of fibers in the vastus lateralis muscles decreases by 50%, whereas the number of motor units in the extensor brevis muscles remains constant from 5 to 50 years of age, decreasing linearly toward zero at 95 years of age (Faulkner et al. 2007). Campbell et al. (1973) reported that the most important factor of wasting and weakness of aging muscles is a reduction in the number of functional motor units. A single motor neuron innervates multiple muscle fibers, and this single neuron and the muscle fibers it innervates is defined as a motor unit. Since the motor unit is the functional unit of muscle contraction, its loss leads to muscle dysfunction. Some of the denervated fibers are incorporated into the remaining motor units by axonal sprouting, and the number of muscle fibers increases in the remaining motor units. As a result of the increased innervation ratio, the ability to control force is reduced (Galganski et al. 1993).

Exercise has been a focus in the prevention of muscle atrophy. It has been confirmed that resistance training effectively prevents muscle wasting and weakness (Fiatarone et al. 1990). There has also been increased interest in nutritional supplementation for the prevention of muscle wasting. For instance, dietary supplementation with amino acids (Kim et al. 2012) or tea catechins (Kim et al. 2013) in combination with moderate-intensity exercise improved walking ability and muscle mass in elderly individuals.

Recent studies have demonstrated that the consumption of whole milk after resistance training can improve body composition and protein synthesis as well as muscle mass in young adults (Elliot et al. 2006, Josse et al. 2010, Wilkinson et al. 2007). However, Kukuljan et al. (2009) reported that the daily consumption of low-fat fortified milk did not enhance the effects of resistance training on skeletal muscle size, strength, or function; thus, results regarding the benefits of milk consumption on muscle mass and strength are controversial. Milk is composed of 3–5% fat, which is distributed in the form of spherical droplets or globules in an emulsion. The triglyceride core of the milk fat globules is surrounded by a thin membrane called the milk fat globule membrane (MFGM), a highly complex structure derived from different protein and lipid components of milk (Cavaletto et al. 2008). Although the biological importance of MFGM remains under study, we recently found that the combination of dietary supplementation with MFGM plus habitual exercise suppressed the aging-associated deterioration of muscle mass and strength via development of the neuromuscular junction (NMJ), the synapse of motor units, in senescence-accelerated mice (Haramizu et al. 2014a). We also demonstrated that dietary MFGM combined with regular exercise improved endurance capacity in mice (Haramizu et al. 2014b).

Therefore, the aim of the present study was to investigate the efficacy of dietary MFGM supplementation plus habitual exercise on physical performance and muscle mass and strength in middle-aged adults. Since a recent epidemiological study in Japan showed that muscle wasting and weakness begins at the age of 50 years (Shimokata et al. 2014), we conducted this randomized, double-blind, placebo-controlled trial of middle-aged Japanese adults.

## Methods

### Subjects

Twenty-two male (50–67 years) and 22 female (50–69 years) healthy volunteer subjects were enrolled in this study. Subjects were excluded from the study if they had coronary heart disease or uncontrolled hypertension or participated in resistance training in their daily life. Written informed consent was obtained from the subjects after being fully informed regarding the details and methods of this study. The study was performed under the supervision of an occupational health physician in accordance with the regulations of the Kao Corporation Ethics Committee for Internal Clinical Studies and in conformity with the Helsinki Declaration.

### Study protocol

A double-blind randomized controlled trial design was used. The randomization procedure was conducted by a person who was not involved in the study, and the subjects and test staff remained unaware of the assignments throughout its duration. The subjects were randomly assigned into one of two groups: one received placebo tablets (placebo group, n = 22: men, n = 11; women, n = 11) and the other received MFGM tablets (MFGM group, n = 22: men, n = 11; women, n = 11). Subjects ingested the MFGM-containing or placebo tablets every day during the 10-week study period and engaged in the exercise training twice per week. Subjects were instructed not to change their daily exercise intensity or diet during the study period. To monitor their daily activities, the subjects wore an electronic pedometer and recorded their energy expenditure throughout their daily activities. To monitor their dairy product intake, they were given record sheets that were collected every 5 weeks on which they recorded the amount of milk and/or dairy products.

### MFGM consumption

The tablets containing 167 mg MFGM/tablet were produced by direct compression of mixtures. The placebo tablet was prepared using whole milk powder instead of MFGM. The placebo tablets had an indistinguishably similar shape, taste, and texture as that of the MFGM tablets. Each subject consumed six MFGM tablets (1 g MFGM/day: equivalent to approximately 600 mL of milk) or the placebo (1 g whole milk powder/day) tablet daily for 10 weeks. On days with exercise training, the subjects were instructed to consume the tablets within 1 hour before the training. On the other days, the subjects were instructed to ingest the tablets during their daily routines.

The MFGM was prepared from buttermilk by filtering and centrifugation, and the MFGM and whole milk powder compositions were analyzed at Japan Food Research Laboratories (Tokyo, Japan). The MFGM composition was 53.4% protein, 24.2% fat, 12.2% carbohydrate, 18.4% phospholipids (4.95% phosphatidylcholine, 5.10% phosphatidylethanolamine, 1.62% phosphatidylinositol, 1.90% phosphatidylserine, 3.81% sphingomyelin, and other phospholipids), 5.1% ash, and 5.1% moisture. The composition of the whole milk powder (Meiji Co., Tokyo, Japan) was 26.8% protein, 26.0% fat, 38.3% carbohydrate, 0.322% phospholipids (0.076% phosphatidylcholine, 0.035% phosphatidylethanolamine, 0.044% phosphatidylinositol, 0.044% phosphatidylserine, 0.067% sphingomyelin, and other phospholipids), 5.8% ash, and 3.6% moisture.

### Exercise training

Each subject underwent exercise training twice weekly on nonconsecutive days for 10 weeks at the designated facility under a physician’s supervision. The exercise consisted of 15-minute sessions each of walking and cycle training at moderate intensity, maintained at approximately 12–14 on the Borg Rating of Perceived Exertion scale (Borg 1982).

### Physical function test

The physical function testing comprised isometric and isokinetic muscle strength as well as side step and chair stand tests. Tests were conducted before and at 5 and 10 weeks of the interventional period. Muscle cross-sectional area (CSA) was measured before and after the 10-week intervention.

#### Anthropometric measurements

Height was measured before the interventional period. Body weight was measured on each visit. Measurements of height and body weight were used to calculate body mass index (kg/m^2^). Blood pressure was measured in the sedentary position after a 5-minute rest at an ambient temperature at 25°C using a mercury manometer.

#### Blood sampling and analysis

Overnight fasting blood was collected for the serum tests from an intermediate vein of the forearm before the following function testing. Serum aspartate aminotransferase (AST), alanine aminotransferase (ALT), glucose, triglyceride, and total cholesterol were analyzed by SRL Inc. (Tokyo, Japan).

#### Knee extension strength

Isometric knee extension strength of each leg was measured using a Force Measurement System for One Leg (T.K.K.5715; TAKEI Scientific Instruments Co., Niigata, Japan) equipped with a tensiometer D (T.K.K.5710e; TAKEI Scientific Instruments Co.) (Goto et al. 2014). The subjects performed two maximal 3-second voluntary contractions at 90° knee flexion with 10 seconds of rest between each attempt. The average muscle peak strength of each leg was calculated as the maximal isometric knee extension strength.

#### Leg extension strength

The isokinetic leg extension strength of each leg was measured using a StrengthErgo 240 (Mitsubishi Electric Co., Tokyo, Japan). Each subject rode on the apparatus and pedaled at maximum effort five times. The peak muscle strength of the right and left legs was calculated as the maximal isokinetic leg extension strength.

#### Side step test

Side step test performance was measured as an indicator of agility and coordination. The subject stood at a center line, stepped 100 cm to the side and touched a line with the closest foot, stepped back to the center, and then stepped 100 cm to the other side and back to the center again for one complete cycle (Sawada et al. 2010). The number of steps performed within 20 seconds was counted.

#### Chair stand test

Chair stand test performance was measured as an indicator of muscular endurance and agility. The subjects stood up and sat down five times with their arms folded in front of their chests as quickly as possible on a firm chair (Jones et al. 1999). The time required to complete five cycles was measured.

#### Muscle CSA

To obtain the CSA of the quadriceps muscles, magnetic resonance imaging of each thigh was obtained using a 1.5 T superconducting system (EXCELART Vantage 1.5 T; Toshiba Medical Systems Co., Japan, Tokyo, Japan). The thigh image obtained 100 mm proximal to the knee joint was utilized for calculation of the CSA using a SCION image (Goto et al. 2011).

#### Surface electromyography (EMG)

Surface EMG signals of the intermediate portion of the right vastus medialis were detected during a knee extension strength measurement with a multiple electrode composed of eight parallel platinum wires (diameter, 1.0 mm; length, 10.0 mm; distance between each electrode, 5.0 mm; Unique Medical, Tokyo, Japan). After preparing the skin with an alcohol swab to reduce impedance, the electrode was installed directly on the skin surface vertical to the underlying muscle fibers and then fixed with stretching rubber. The EMG signal was amplified using a differential bioelectric amplifier (AB-611 J; Nihon-Kohden Co., Tokyo, Japan). The frequency range of the amplifier was set at 5–1000 Hz. After the A/D conversion, the root mean square (RMS) amplitude and motor unit recruitment level were calculated using a time epoch of 1 second for the EMG. Muscle fiber conduction velocity (MFCV) was estimated using cross-correlation, and signals with correlation coefficients of ≥0.8 were adopted to calculate the MFCV (Mito et al. 2006).

### Statistical analysis

All evaluation variables are presented as means ± SEM. A 2-way ANOVA (analysis of variance) using the time point as the repeated measure and the group as the non-repeated measure was performed. When a significant group-by-time interaction was observed, an unpaired *t*-test was used for intergroup comparison at each time point, and the Bonferroni post hoc test was used for intragroup comparison during the interventional period. In a separate analysis, change in percentage from baseline to 10 weeks was evaluated using the unpaired *t*-test for intergroup comparison and the paired *t*-test for intragroup comparison. For the statistical analysis, STATVIEW for WINDOWS software (SAS Institute Inc., Cary, NC, USA) was used. All P values were two-tailed, and those <0.05 were considered statistically significant.

## Results

### Subjects and compliance

The subjects’ baseline characteristics were similar between groups (Table 1). The subjects tolerated the intervention protocol well, and the overall compliance rate was >98%. There was no report of adverse side effects from the test tablets. Nine subjects (placebo = 6, MFGM = 3) were unable to complete the study after randomization because of a lack of motivation for training (n = 5) and private reasons unrelated to the intervention (n = 4). Energy expenditure through daily activities calculated using an electronic pedometer during weeks 0, 5, and 10 in the MFGM group were 1639 ± 56, 1724 ± 61, and 1739 ± 60 kcal/day; in the placebo group, they were 1749 ± 57, 1845 ± 66, and 1861 ± 77 kcal/day, respectively. The average daily intake of milk and dairy products such as yogurt, cheese, butter, and milk beverages during weeks 0, 5, and 10 in the MFGM group were 147 ± 22, 135 ± 25, and 166 ± 30 g/day; in the placebo group, they were 143 ± 27, 138 ± 25, and 155 ± 26 g/day, respectively. No significant inter- or intragroup differences were noted in energy expenditure by daily activities and dairy intake. Likewise, there were no major changes in exercise or diet volume during the study period (other data not shown).

**Table 1:** **Subjects’ baseline characteristics**

	**Total**		**Women**		**Men**	
	**Placebo**	**MFGM**	**Placebo**	**MFGM**	**Placebo**	**MFGM**
	**(n = 22)**	**(n = 22)**	**(n = 11)**	**(n = 11)**	**(n = 11)**	**(n = 11)**
Age, years	59.0 ± 1.4	57.6 ± 1.2	59.5 ± 2.0	57.9 ± 1.7	58.5 ± 2.1	57.3 ± 1.7
Height, cm	160.7 ± 1.8	161.2 ± 2.1	153.9 ± 1.6	153.7 ± 2.1	167.5 ± 1.2	168.6 ± 1.9
Body weight, kg	62.3 ± 2.2	59.9 ± 2.3	55.2 ± 1.7	53.8 ± 3.0	69.5 ± 2.6	66.1 ± 2.2
Body mass index	24.0 ± 0.6	23.0 ± 0.7	23.3 ± 0.7	22.8 ± 1.3	24.8 ± 1.0	23.3 ± 0.7
Knee extension, kg	34.0 ± 3.1	36.4 ± 3.1	23.4 ± 1.4	25.5 ± 2.6	44.7 ± 4.1	47.4 ± 3.0
Leg extension, Nm	100.8 ± 7.8	101.1 ± 8.4	70.7 ± 5.5	71.5 ± 6.5	130.9 ± 6.9	130.7 ± 8.9
Systolic BP, mmHg	122.3 ± 3.6	124.0 ± 4.8	118.1 ± 4.1	118.3 ± 3.0	126.5 ± 5.9	129.7 ± 9.0
Diastolic BP, mmHg	76.4 ± 2.9	76.4 ± 2.8	69.8 ± 3.1	69.0 ± 2.3	82.9 ± 4.0	83.7 ± 4.2

MFGM, milk fat globule membrane; BP, blood pressure.

Values are means ± SEM. Measurements did not differ between groups.

### Effect on anthropometric values and serum variables

No overall changes in body weight or blood pressure were observed during the interventional period (data not shown). There were no clinically significant changes in fasting serum aspartate aminotransferase, alanine aminotransferase, glucose, triglyceride, and total cholesterol level during the 10-week intervention (Table 2). Regarding the serum variables, mean values did not deviate from the Japanese standard values (SRL Inc. homepage) throughout the interventional period.

**Table 2:** **Values of serum variables before and after the intervention**

		**Placebo**	**MFGM**	**ANOVA (G × T)**
				***P*** **-value**
Aspartate aminotransferase, IU/L	baseline	24.5 ± 1.82	22.1 ± 0.98	0.591
	5 weeks	25.1 ± 2.53	22.3 ± 0.77	
	10 weeks	24.1 ± 1.52	22.7 ± 1.06	
	Δ 10 weeks (%)^a^	1.0 ± 5.3	5.1 ± 5.0	
Alanine aminotransferase, IU/L	baseline	25.1 ± 4.33	20.1 ± 2.02	0.674
	5 weeks	23.7 ± 4.49	18.8 ± 2.03	
	10 weeks	22.9 ± 2.79	19.7 ± 2.38	
	Δ 10 weeks (%)^a^	- 0.1 ± 7.3	1.7 ± 6.6	
Glucose, mg/dl	baseline	93.3 ± 2.01	90.4 ± 2.13	0.827
	5 weeks	93.4 ± 2.81	90.5 ± 2.21	
	10 weeks	90.4 ± 2.22	88.6 ± 1.96	
	Δ 10 weeks (%)^a^	- 3.0 ± 1.8	- 1.8 ± 1.2	
Triglyceride, mg/dl	baseline	132 ± 12.7	104 ± 9.4	0.536
	5 weeks	103 ± 10.2	89 ± 6.4	
	10 weeks	109 ± 10.9	95 ± 8.6	
	Δ 10 weeks (%)^a^	- 12.3 ± 6.7	- 2.6 ± 8.9	
Total cholesterol, mg/dl	baseline	206 ± 9.2	219 ± 9.8	0.190
	5 weeks	210 ± 10.8	221 ± 9.8	
	10 weeks	211 ± 9.9	212 ± 7.5	
	Δ 10 weeks (%)^a^	2.9 ± 2.3	- 2.0 ± 2.0	

MFGM, milk fat globule membrane; ANOVA, analysis of variance.

Values are means ± SEM of 16 (placebo group [women, n = 7; men, n = 9]) and 19 (MFGM group [women, n = 11; men, n = 8]) subjects.

^a^The value is the percentage change from the baseline to 10 weeks. Repeated-measures ANOVA for the group-by-time interaction (G × T) during the interventional period.

### Effect on muscle strength, physical function, and muscle CSA

After 10 weeks of intervention, a significant group-by-time interaction was found for the side step test and muscle CSA (Table 3). No significant change was noted in the placebo group. In the MFGM group, side step score and muscle CSA were significantly greater at 10 weeks than at the baseline. In addition, the changes from the baseline of the side step score and muscle CSA were significantly higher in the MFGM group than in the placebo group at 10 weeks. No significant group-by-time interaction was observed in the knee and leg extension strength and chair stand scores, whereas they were significantly increased from baseline to 10 weeks in the MFGM group. A significant time effect was found for the knee extension, leg extension, and chair stand tests. No significant changes were noted in the placebo group during the interventional period. In contrast, the knee extension and leg extension strength, and chair stand scores were significantly improved at 10 weeks compared with the baseline in the MFGM group.

**Table 3:** **Muscle strength, physical functions, and muscle cross-sectional area values before and after the intervention**

		**Placebo**	**MFGM**	**ANOVA (G × T)**
				***P*** **-value**
Knee extension, kg	baseline	32.2 ± 3.1	34.1 ± 3.2	0.642
	5 weeks	33.3 ± 3.1	35.9 ± 3.6	
	10 weeks	33.8 ± 3.2	37.1 ± 3.8	
	Δ 10 weeks (%)^a^	6.0 ± 4.0	9.7 ± 3.4^d^	
Leg extension, Nm	baseline	102.9 ± 9.6	97.4 ± 8.6	0.567
	5 weeks	108.4 ± 11.0	102.0 ± 8.8	
	10 weeks	107.1 ± 10.6	103.8 ± 8.4	
	Δ 10 weeks (%)^a^	4.2 ± 2.6	6.4 ± 2.0^d^	
Side step test, times	baseline	32.5 ± 1.6	32.3 ± 1.5	0.047
	5 weeks	33.5 ± 1.6	34.3 ± 1.5^b^	
	10 weeks	33.3 ± 1.6	34.4 ± 1.5^b^	
	Δ 10 weeks (%)^a^	2.4 ± 1.3	6.8 ± 1.6^c d^	
Chair stand test, sec	baseline	5.7 ± 0.4	5.9 ± 0.4	0.590
	5 weeks	5.6 ± 0.3	5.5 ± 0.4	
	10 weeks	5.4 ± 0.4	5.3 ± 0.4	
	Δ 10 weeks (%)^a^	- 4.6 ± 3.3	- 10.4 ± 2.3^d^	
Muscle CSA, cm^2^	baseline	129.2 ± 7.4	116.2 ± 5.7	0.025
	10 weeks	129.4 ± 7.0	120.6 ± 5.7^b^	
	Δ 10 weeks (%)^a^	0.4 ± 1.0	4.0 ± 1.1^c, d^	

MFGM, milk fat globule membrane; ANOVA, analysis of variance; CSA, cross-sectional area.

Values are means ± SEM of 16 (placebo group [women, n = 7; men, n = 9]) and 19 (MFGM group [women, n = 11; men, n = 8]) subjects.

^a^The value is the percentage change from the baseline to 10 weeks. Repeated-measures ANOVA for the group-by-time interaction (G × T) during the interventional period. ^b^There was a significant difference from the baseline during the interventional period, as determined using the Bonferroni test (*P* < 0.05). ^c^There was a significant difference between the two groups in results of the unpaired *t*-test (*P* < 0.05).^d^There was a significant difference from the baseline to 10 weeks in results of the paired *t*-test (*P* < 0.05).

### Effect on EMG parameters (RMS and MFCV)

RMS and MFCV values were derived from the vastus medialis muscles during isometric maximal voluntary contraction (Table 4). A significant group-by-time interaction was found for MFCV during the interventional period. In the placebo group, the MFCV significantly decreased at 10 weeks compared with the baseline. The MFCV and the change from the baseline were significantly higher in the MFGM group than the placebo group at 10 weeks. A significant time effect was found for RMS, an indicator of motor unit recruitment. Whereas no significant difference was noted between the groups, the RMS was significantly increased at 10 weeks compared with the baseline in the MFGM group.

**Table 4:** **Root mean square (RMS) amplitude of electromyogram (EMG) and muscle fiber conduction velocity (MFCV) values during maximal voluntary contraction in the vastus medialis**

		**Placebo**	**MFGM**	**ANOVA (G × T)**
				***P*** **-value**
RMS, mV	baseline	0.133 ± 0.017	0.145 ± 0.019	0.778
	5 weeks	0.156 ± 0.016	0.172 ± 0.019	
	10 weeks	0.153 ± 0.014	0.175 ± 0.022	
	Δ 10 weeks (%)^a^	27.3 ± 10.6^d^	27.1 ± 10.4^d^	
MFCV, m/sec	baseline	5.91 ± 0.39	5.77 ± 0.32	0.046
	5 weeks	5.02 ± 0.48	5.43 ± 0.22	
	10 weeks	4.17 ± 0.45^b^	5.58 ± 0.39^c^	
	Δ 10 weeks (%)^a^	- 27.2 ± 8.2^d^	1.1 ± 8.9^c^	

MFGM, milk fat globule membrane; ANOVA, analysis of variance.

Values are means ± SEM of 16 (placebo group [women, n = 7; men, n = 9]) and 19 (MFGM group [women, n = 11; men, n = 8]) subjects for RMS and 12 (placebo group [women, n = 5; men, n = 7]) and 16 (MFGM group [women, n = 10; men, n = 6]) subjects for MFCV, respectively.

^a^The value is the percentage change from the baseline to 10 weeks. Repeated-measures ANOVA for the group-by-time interaction (G × T) during the interventional period. ^b^There was a significant difference from the baseline during the interventional period, as determined using the Bonferroni test (*P* < 0.05). ^c^There was a significant difference between the two groups in results of the unpaired *t*-test (*P* < 0.05). ^d^There was a significant difference from the baseline to 10 weeks in results of the paired *t*-test (*P* < 0.05).

## Discussion

To our knowledge, the present study is the first to demonstrate the combination of MFGM intake and habitual exercise improved physical performance such as agility in middle-aged adults.

Habitual exercise alone (placebo group) did not exhibit significant effects on physical performance or muscle strength in this study. In a review, Lynch et al. (2007) suggested that resistance training is the most effective exercise for slowing muscle wasting and maintaining or improving muscle strength, whereas moderate aerobic training is effective for increasing cardiovascular fitness. The lack of efficacy of habitual exercise alone may be due to the moderate intensity of the exercise program (Borg 1982) used in our study. Interestingly, when the exercise program at moderate intensity for 10 weeks was combined with dietary supplementation of MFGM, the side step performance, which requires agility and coordination in the lower limbs, and the quadriceps muscles CSA significantly increased even though there was no change in whole body composition (BMI). The subjects performed the exercise training mainly using lower limbs (walking and cycle exercise) in this study. These results may indicate that the MFGM supplementation boost the beneficial effects of moderate exercise on the trained muscles topically. The results were compatible with those in our previous studies, which demonstrated that dietary MFGM plus habitual exercise improved physical performance and muscle mass and strength in mice (Haramizu et al. 2014a, b).

Kim et al. (2012) showed that the supplementation of amino acids (6 g/d) combined with habitual exercise improved muscle mass and strength in women with sarcopenia. In this study, amino acid (including protein) supplementation from MFGM or whole milk powder (1 g/d) was only 0.534 g and 0.268 g daily, respectively. Our previous study in mice suggested that phospholipids, sphingomyelin in particular, might be one of the active components of MFGM promoting neuromuscular development (Haramizu et al. 2014a). Therefore, the contribution of proteins or amino acids in MFGM to the beneficial effects on physical performance and muscle function seems only slight.

Dietary MFGM plus habitual exercise improved the side step score, an indicator of agility, the ability to make specific actions and movements both quickly and accurately (Vescovi 2006). Changes in muscle contraction velocity and agility in aging are highly related to the motor neuron or MFCV (Campbell et al. 1973). Our previous study suggested that a major transcriptomic effect of dietary MFGM plus habitual exercise in skeletal muscle was “nervous system development” in senescence-accelerated mice (Haramizu et al. 2014a). Dietary supplementation with MFGM combined with habitual exercise increased the expression of Dok-7 (Haramizu et al. 2014a), one of the key molecules of NMJ formation (Okada et al. 2006). The NMJ plays an important role in the conduction of action potentials and neuromuscular maintenance. It has been reported that the deterioration of NMJ may be responsible for aging-related muscle dysfunction (Shigemoto et al. 2010). Valdez et al. (2010) recently demonstrated that aging-related morphologic changes of the NMJ are affected by dietary restriction and voluntary exercise in an aging model, and these findings suggest that nutritional and exercise intervention may be effective for neuromuscular maintenance, including NMJ formation. Even though a limitation of this study was that we did not directly examine NMJ function and its related molecules, our EMG analysis showed that habitual exercise increased RMS, an indicator motor unit recruitment, and dietary MFGM plus habitual exercise increased MFCV, suggesting increased fast-type motor unit recruitment (Farina et al. 2004) and/or maintenance of the fast/slow ratio of muscle fiber type with treatment (Sadoyama et al. 1988). A higher MFCV after 10-week treatment indicates that dietary MFGM plus habitual exercise increased the recruitment of motor unit populations (Houtman et al. 2003). Therefore, we speculate that dietary supplementation with MFGM combined with habitual moderate-intensity exercise improved neuromuscular function in middle-aged adults.

Increased muscle strength during the early stage of exercise training is attributed to improvements in the nervous system rather than the muscular system (Moritani & DeVries 1979). To better understand age-related muscle wasting and weakness, the importance of focusing on neuromuscular functions such as α-motor neurons or motor unit activity has been recognized (Roos et al. 1997, Manini & Clark 2012). Significant NMJ fragmentation and muscular denervation with aging occur more predominantly in fast- than slow-twitch muscle fibers (Balice-Gordon 1997). Therefore, fast-twitch muscle fiber-predominant denervation accelerates the type-switching to slow-twitch. The combination of exercise and MFGM may have promoted the formation of fast-type NMJ.

The present study demonstrates that dietary MFGM plus habitual exercise enhances both agility and muscle CSA in middle-aged adults. Furthermore, the results suggest that increased motor unit recruitment and maintenance of fast-type muscle fibers play significant roles in the beneficial effects of treatment on physical performance and muscle CSA in middle-aged adults. The findings will increase our understanding of the role of dietary supplementation in counteracting the age-related decline in muscle strength and physical function. Studies are in progress to clarify the mechanism underlying the beneficial actions of MFGM plus habitual exercise on motor units. Nutritional intervention combined with exercise training might be important for enhancing quality of life in elderly individuals.

## Conclusion

The present study provides evidence that dietary MFGM supplementation plus habitual exercise can improve physical performance, such as agility, in middle-aged Japanese adults. The beneficial effects of MFGM plus exercise treatment may be due to the increase in the motor unit maintenance of fast-twitch muscle fibers.
